# Improvement in Depressive Symptoms Is Not Associated with the Severity of Autobiographical Amnesia Following Electroconvulsive Therapy—A Preliminary Report from Naturalistic Prospective Observational Study

**DOI:** 10.3390/jcm14217663

**Published:** 2025-10-28

**Authors:** Albert Stachura, Stefan Sawicki, Łukasz Święcicki

**Affiliations:** 1 Department of Methodology, Medical University of Warsaw, ul. Żwirki i Wigury, 02-061 Warsaw, Poland; 2 2nd Department of Psychiatry, Institute of Psychiatry and Neurology, ul. Sobieskiego 9, 02-957 Warsaw, Poland

**Keywords:** electroconvulsive therapy, ECT, depressive disorder, cognitive function, autobiographical memory

## Abstract

**Background/Objectives**: Electroconvulsive therapy (ECT) is used for treatment-resistant depression (TRD). Despite high effectiveness, its most prevalent side effect is memory loss, particularly autobiographical memory deficits. Some patients and physicians might associate post-ECT improvement in depressive symptoms with a higher risk of autobiographical amnesia or even consider this side effect ECT’s mechanism of action. Here, we aimed to study the association between improvement in depressive symptoms and the severity of autobiographical amnesia, as well as identify factors associated with the degree of memory loss. **Methods**: In this prospective naturalistic observational pilot study, we included 20 patients who underwent ECT for TRD. Attending psychiatrists decided on the electrode placement. Electrical dosage was based on the seizure-titration procedure. Depressive symptoms (Hamilton Depression Scale-21) and autobiographical memory (AMI-SF) were assessed before and after the full course of ECT. The correlation between symptomatic improvements and severity of memory loss was studied using Spearman’s correlation. Demographic and clinical baseline data were examined to look for associations with the decline in autobiographical memory. **Results**: Symptomatic improvement was not correlated with autobiographical memory loss (r = −0.14, *p* = 0.58) or any of its domains. Suicidal ideation at baseline was associated with a stronger decline in autobiographical memory (r = −0.53, *p* = 0.016). Patients treated with bilateral electrode placement had worse amnesia than those treated with right unilateral treatment, though the difference was not significant (MD = −17.4 vs. −13.1, *p* = 0.2). **Conclusions**: ECT improved depressive symptoms irrespective of autobiographical amnesia severity. Patients with suicidal ideation at baseline might experience worse post-ECT amnesia than those without.

## 1. Introduction

Approximately 1.4 million people are treated annually with electroconvulsive therapy (ECT) with treatment-resistant depression being the most common indication in Western countries [1]. Remission rates for this therapy range between 60% and 70% [2]. It also rapidly reduces suicidal ideation and improves quality of life [3]. Despite high efficacy, it is associated with retrograde autobiographical amnesia (RAA) [4]. This refers to difficulties in retrieving memories of events or facts acquired before the treatment [5]. More than half of the treated patients spontaneously report experiencing some sort of memory deficits following ECT [6]. This phenomenon should be distinguished from cognitive impairments, which might be present before treatment and are associated with depressive disorder itself. Attention, processing speed and cognitive inhibition improve after ECT and are parallel to symptom reduction [7]. Another study showed that most cognitive functions improve or are unaffected by ECT with remitters having better self-rated memory, anterograde verbal memory and category verbal memory compared with non-remitters [8]. Autobiographical memory on the other hand declines after ECT and the effect size is large [4]. Long-term data on RAA are lacking or of poor quality (i.e., follow-up drop-out rates in some studies are as high as 60%). Based on the available evidence, it is not feasible to predict whether a particular patient will have lasting or transient (i.e., resolving within months) personal memory deficits. The qualitative literature suggests that patients may experience long-lasting autobiographical memory deficits, which might have a negative impact on their daily functioning [4].

Data from the qualitative literature suggest that despite decades of research on RAA, its nature, severity and impact on patient’s lives remain uncertain [4]. Patients who improve after ECT seem to be more accepting of treatment-induced memory deficits as qualitative data suggest [4]. This underscores the importance of considering response predictors (i.e., those presented in the DEC trial) [9] when qualifying a patient for ECT. Some are concerned that ECT’s effectiveness might be somehow linked to its capacity to elicit memory-related harms [4]. Understanding the relationship between the severity of RAA and the degree of symptomatic improvement might be useful to help guide shared decision-making process, as some patients might be afraid that symptomatic improvements can only be achieved at the cost of personal memory loss. Presenting the data confirming or undermining this link could give a patient more confidence when selecting treatment options.

Here, we presented the results of an interim analysis, in which we aimed to study if ECT-mediated improvements in depressive symptoms were correlated with the severity of RAA. Our hypothesis was that there might be a weak or no association between these two phenomena. Furthermore, we checked if any patient-specific characteristics might have been linked to the degree of post-ECT autobiographical memory loss. Our recent meta-analysis showed that patients with severe depression may experience more RAA than those with moderate depression, though the difference was not statistically significant [4]. We also looked at differences in the severity of RAA between patients treated with bilateral vs. unilateral ECT. Based on the current evidence, we hypothesized that bilateral treatment would cause more severe RAA than right unilateral treatment.

## 2. Materials and Methods

### 2.1. Study Design

This was a prospective naturalistic observational study, approved of by the Bioethical Committee by the Medical University of Warsaw, approval number KB/112/2022. All patients included in the study provided informed consent to participate. The study was conducted at the Institute of Psychiatry and Neurology, Warsaw, Poland. Recruitment, treatment and data collection occurred between February 2023 and December 2024.

All potentially eligible inpatients treated in the 2nd Psychiatric Clinic were consecutively approached and asked for their consent to participate in the study. Patients were included if they were adult (aged 18 or above), were diagnosed with treatment-resistant depressive disorder (uni- or bipolar) and were referred for electroconvulsive therapy by their treating physician. Exclusion criteria were as follows: (1) inability to provide informed consent or a lack thereof, (2) severe dementia or a comorbid disease severely impairing one’s cognitive functions. The treatment resistance criteria were used in accordance with the definition postulated by the EMA and FDA, i.e., inadequate response to a minimum of two antidepressants despite adequacy of the treatment trial and adherence to treatment [10].

The exposure and comparators were right unilateral (RUL) ECT and bilateral (BL) ECT. These two treatments were treated as exposures in the study context because the authors did not interfere with the attending physician’s and the ECT team’s decision on the electrode placement. During the first session, the seizure threshold (ST) for each patient was identified using the titration method [11]. In the case of RUL ECT, for subsequent sessions the dosage was 6xST and for BL ECT it was 2.5xST. Electroconvulsive therapy was administered using Thymatron^®^ System IV (Somatics LLC, Venice, FL, USA). For anesthesia, ethomidate and succinylcholine were used. Treatment with mood stabilizers was discontinued before ECT to avoid seizure threshold elevation or an increased risk of delirium in the case of lithium treatment [12]. No medications were added during ECT and the remaining medications were either maintained or discontinued.

### 2.2. Psychometric Evaluation and Data Collection

The primary outcome of interest was change in autobiographical memory as measured with the Columbia University Autobiographical Memory Inventory, i.e., CUAMI-SF (AMI-SF) [13]. The tool consists of 30 questions within 6 segments, and the maximum total score is 60. The Polish version utilized in this study was prepared by the authors (AS and SS). First, SS translated the tool into Polish and the translation was double-checked by AS. Secondary outcomes were changes in depressive symptoms as measured with the Hamilton Depression Severity Scale (HAM-D-21) [14] and domains of autobiographical memory as suggested by Semkovska et al. [15]. They distinguished three components: semantic (a sum of points scored on themes Family Member and Last Employment), episodic extended (total score on themes Last Major Trip and Last Physical Illness) and episodic specific (total score on themes Last New Year’s Eve and Last Birthday). Testing was performed within three days before the first ECT session and again within 5 days after the last ECT session. Additionally, sociodemographic and clinical data were collected: age, sex, presence of psychotic symptoms or suicidal ideation, depression type, number of previous episodes, duration of current episode, education, occupational status, presence of comorbid personality disorder, ECT type, number of sessions and mean charge.

### 2.3. Sample Size Calculation

Sample size calculation was based on the assumed difference in total AMI-SF decline between patients treated with RUL and BL ECT. G*Power 3.1 was used for the calculations using a *t*-test and assumed effect size of 0.95, statistical power of 95% and statistical significance threshold of 0.05 [16]. Assuming a 20% drop-out rate at post-treatment assessment, the final sample size of 72 was calculated. Due to limited access to patients requiring ECT and recruitment challenges, here we present an interim analysis exploring associations between retrograde autobiographical amnesia and depression improvement following ECT.

### 2.4. Statistical Analysis

Descriptive statistics included means ± standard deviations for continuous variables and numbers with percentages for categorical data, unless otherwise indicated. For continuous variables, we first assessed data distribution with QQ plots and used parametric tests (*t*-test) for normally distributed data and nonparametric tests (Wilcoxon, Mann–Whitney *U* tests) for non-normally distributed data. To test correlations, we calculated Spearman’s correlation coefficients with *p*-values. The threshold for statistical significance was set at 0.05 unless otherwise indicated. In the case of multiple comparisons, we applied Hochberg’s correction. All analyses were performed using R version 4.5.0.

## 3. Results

The sociodemographic and clinical data of included patients are presented in Table 1. Most of them were females (80%), less than half of them had psychotic symptoms or suicidal ideations. Most were diagnosed with severe unipolar depression lasting 10 months on average. Nearly half of the patients had experienced more than four previous depressive episodes. Three patients were diagnosed with personality disorder. Most study participants underwent unilateral ECT with a mean of 11 sessions.

We noted autobiographical memory decline in all patients as measured with AMI-SF. Those treated with bilateral ECT suffered from more autobiographical amnesia than those treated with unilateral ECT (Table 2), though the difference could be attributable to chance (*p* = 0.02). A similar trend was observed across all tool domains (semantic, episodic extended and episodic specific). Less-severe memory loss was recorded for the semantic component (MD = −2.3 and −4 for unilateral and bilateral ECT, respectively), than for the episodic-extended (MD = −4.8 and −7.4) or episodic-specific (MD = −5.7 and −6) components.

**Figure 1:**
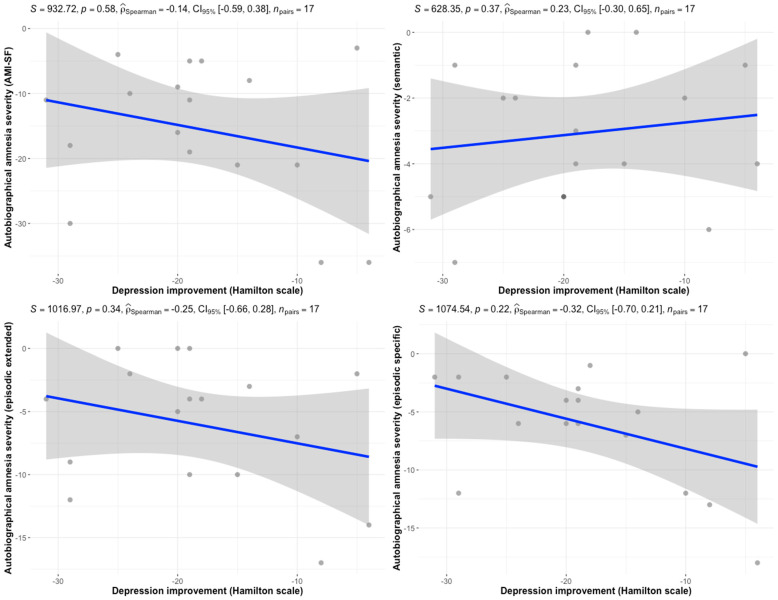
Correlations between depression improvements and autobiographical amnesia severity by AMI-SF domains.

Importantly, improvement in depressive symptoms was not correlated with either total AMI-SF score (r = −0.14, *p* = 0.58) or any of the tool domains (Figure 1).

Autobiographical amnesia was not correlated with most sociodemographic or clinical variables (Table 3). Only suicidal ideation at baseline was linked to a decline in total AMI-SF (r = −0.53, *p* = 0.016), as well as a semantic memory domain (r = −0.49, *p* = 0.028), indicating a stronger memory deficit in patients reporting suicidal ideation before ECT.

## 4. Discussion

We found that bilateral ECT is associated with more severe autobiographical amnesia than unilateral ECT, though the differences were not statistically significant. We did not identify a strong correlation between the treatment’s harm and effectiveness, as measured with the Hamilton Depression Severity Scale. The sole variable associated with a stronger memory deficit was patients’ suicidal ideation at baseline, though the correlation was not significant for episodic components of autobiographical memory. This correlation was not statistically significant after applying the Hochberg correction.

This was a preliminary report of a prospective naturalistic study, and it is unsurprising that despite observing a signal difference between BL and RUL ECT, the effect was not statistically significant. This could be explained by an insufficient sample size and, consequently, a higher variance in data, both of which contribute to lower levels of statistical significance. In our systematic review, we showed that BL ECT causes more severe acute autobiographical amnesia than RUL ECT within 10 days post-treatment with a modest effect size [4]. This is in line with previous meta-analytic research on this topic [17]. In our previous work, we found that pulse width or dosing protocol might play a role in the severity of autobiographical amnesia; however, these variables were not studied here [4]. In all cases, seizure threshold-based protocol was applied and most patients had ECT using brief pulses.

As memory deficits are frequently reported by patients undergoing ECT [4], it has been speculated that said deficits might explain some of the treatment’s effectiveness, acting as its mechanism of action [18]. Frais argued that ECT might help patients unlock the ‘super’ autobiographical memory system, which would allow them to make sense of their experiences and attribute meaning to past events, hence recovering the ‘self’. This hypothesis, however, is at odds with testimonies of some patients who said they had lost parts of their identity following ECT and struggled to rebuild it [4]. Though cognitive abilities improve in depressed patients after ECT, as mentioned before [7], the nature of autobiographical memory loss still remains elusive. Meta-analytic evidence shows that ECT causes more RAA than other forms of anti-depressive treatments and therefore associating ECT with only cognitive improvement does not hold [4]. Some patients undergoing treatment raised concerns that this therapy’s benefits and harms co-occur and thus may be causally related [19,20]. Our results challenge these assumptions as we have shown no strong correlation between the improvement in depressive symptoms and the severity of autobiographical amnesia. In fact, we showed weak and insignificant negative correlations between total AMI-SF score or its episodic components and improvement of depressive symptoms. This would imply a correlation between an improvement in depressive symptoms and less severe autobiographical amnesia. On the other hand, the semantic component was positively correlated with a change in HAM-D-21, implying that a decline in this aspect of personal memory may be correlated with an improvement in depressive symptoms. In any case, based on our results, a sample size of between 75 (for r = −0.32) and 399 (for r = −0.14) would be required to detect a significant correlation. These are low correlation coefficients, which, even if rendered statistically significant, are unlikely to explain the degree of symptomatic improvement following ECT. To our knowledge, this is the first report providing such an analysis on an individual participant’s level. These findings are supported by the work by Anderson et al., who showed that the severity of autobiographical amnesia does not differ between remitters and non-remitters at four-month follow up after ECT [8]. This suggests that the degree of autobiographical amnesia is not strongly linked to acute response to ECT nor does it help to predict the long-term outcome. Therefore, identifying individuals at a higher risk of clinically significant amnesia is challenging and not necessarily associated with the treatment response. Importantly, patients who improve after ECT tend to find harms more acceptable and probably are less likely to report them [4]. It means that unless clinicians ask directly about autobiographical amnesia, they might fail to detect it in some patients.

As memory loss is prevalent among patients who underwent ECT, identifying factors associated with more severe amnesia is key to planning post-treatment care and improving adequate qualifications for ECT. Here, we found that suicidal ideation at baseline was correlated with a stronger decline in autobiographical memory, though this finding is rendered insignificant after correcting for multiple comparisons using the Hochberg method. Previous research indicated that impairments in verbal fluency might be linked to worse autobiographical amnesia [21]; however, we did not find studies reporting correlations between baseline patient’s characteristics and memory loss. A few studies looked at orientation time and found age, female sex and pulse width to be associated with longer immediate post-treatment recovery time [21,22] but there seems to be no link between orientation time and retrograde amnesia severity. A recent systematic review showed that autobiographical memory is less specific in patients with a history of attempted suicide compared with those without [23]. Authors suggested that impaired memory might prevent suicidal individuals from solving current problems using knowledge of past experiences and they might find it challenging to envision a more positive future. Another study showed that suicidal ideation and violent daydreaming were associated with impaired everyday memory retrieval and encoding [24]. These data suggest that individuals with suicidal ideation may have altered memory function, and it might be possible that they are also more prone to developing autobiographical memory deficits as suggested here. This finding requires replication and further study in larger clinical samples.

This study is limited by a small sample size due to challenges in recruitment and limited access to patients requiring ECT. Though small, our sample should be sufficient to visualize strong associations or correlations. Assuming 80% power and a statistical threshold of 0.05, any correlation equivalent to or stronger than r = 0.63 should be statistically significant. A lack of positive findings indicates that even if such a link exists, it might be small or moderate and arguably not highly clinically significant. The robustness of the comparison between BL and RUL treatments was limited by an unequal distribution of patients between the two groups. It was because, in our clinic, most patients undergo RUL ECT for depression to minimize potential side effects. We wanted to use a naturalistic study design so that our report reflects the clinical reality. The findings are also limited by the utility of the tool used to measure autobiographical memory. It has been criticized for focusing on events, which might not be equally relevant to all patients [25]. Moreover, the Polish version was not validated and the translation procedure used in this study might have been imperfect. Indeed, the findings of our qualitative meta-synthesis suggested that patients might experience functional impairment due to autobiographical amnesia (and realize its impact on daily life) only after coming back home and interacting with other people [4]. Nevertheless, AMI-SF is currently the most commonly used and validated tool measuring autobiographical amnesia specifically after ECT, hence our choice to select it for this study. Another limitation was that we did not adjust for potential confounders when examining the link between RAA and improvements in depressive symptoms or RAA and other clinical or sociodemographic data. Given a limited sample size, such adjustments would not be feasible and could lead to potential overfitting of the model. Therefore, larger studies are needed to reassess the hypotheses presented in this study.

## 5. Conclusions

An improvement in depressive symptoms after ECT is not strongly correlated with the degree of autobiographical amnesia, though a weak-to-moderate association cannot be excluded, given the small sample size. Suicidal ideation at baseline might be linked with worse memory loss during treatment, though this finding requires replication in larger samples.

## Figures and Tables

**Table 1:** Characteristics of study participants.

Variable	Sample Size (*n* = 20) Mean ± SD or *n* (%)
Age (years)	49.1 ± 20.2
Sex	
Male	4 (20)
Female	16 (80)
Psychotic symptoms	7 (35)
Suicidal ideation	8 (40)
Depression type	
Unipolar	14 (70)
Bipolar	6 (30)
Number of previous depressive episodes	
None	4 (20)
1–3	7 (35)
>4	9 (45)
Duration of current episode (months)	10.3 ± 8.6
Education	
Primary	1 (5)
Secondary	6 (30)
Higher	13 (65)
Occupational status	
Unemployed	2 (10)
Employed	10 (50)
Disability pension	3 (15)
Retired	5 (25)
Any personality disorder	3 (15)
Baseline depression severity score	27.2 ± 6.6
Baseline AMI-SF score	48.2 ± 7.2
ECT type	
Unilateral	15 (75) *
Bilateral	4 (20)
Mixed placement	1 (5)
Number of ECT sessions	10.8 ± 2.3
Mean charge per session (mC)	387.9 ± 187.1

* One patient underwent ECT using ultrabrief pulses. SD—standard deviation.

**Table 2:** Autobiographical amnesia severity by electrode placement.

	RUL (*n* = 15)			BL (*n* = 5)			*p*-Value
	Pre	Post	MD (95% CI)	Pre	Post	MD (95% CI)	
AMI-SF total	49.3 ± 6.1	36.2 ± 10.8	−13.1 (−18.8 to −7.5)	44.8 ± 9.8	27.4 ± 9.7	−17.4 (−31.1 to −3.7)	0.2
Semantic memory	15.3 ± 2.9	13 ± 3.1	−2.3 (−3.5 to −1.1)	14.4 ± 2.6	10.4 ± 1.5	−4 (−5.9 to −2.0)	0.1
Episodic extended memory	15.7 ± 4.3	10.9 ± 5.3	−4.8 (−7.3 to −2.3)	13.6 ± 4.3	6.2 ± 5.6	−7.4 (−15 to 0.23)	0.33
Episodic specific memory	18.3 ± 1.8	12.6 ± 5.2	−5.7 (−8.4 to −2.9)	16.8 ± 5.2	10.8 ± 5.3	−6 (−11.4 to −0.7)	0.72

**Table 3:** Correlations between participants’ characteristics and autobiographical amnesia severity by AMI-SF domains.

	AMI-SF		Semantic Memory		Episodic Extended		Episodic Specific	
Variable	r	*p*-Value	r	*p*-Value	r	*p*-Value	r	*p*-Value
Age	0.21	0.38	0.06	0.79	0.19	0.4	0.14	0.57
Episode duration	0.11	0.67	0.19	0.42	0	0.97	0.2	0.4
Baseline depression severity	−0.28	0.28	−0.29	0.26	−0.16	0.54	−0.06	0.81
Number of sessions	−0.25	0.29	−0.19	0.41	−0.32	−0.17	−0.03	0.91
Sex	−0.28	0.23	−0.01	0.96	−0.33	0.16	−0.35	0.13
Psychotic symptoms	−0.17	0.46	−0.4	0.08	0.1	0.67	−0.08	0.73
Depression type	−0.24	0.31	−0.13	0.57	−0.18	0.44	−0.12	0.6
Suicidal ideation	−0.53	0.016 *	−0.49	0.028 *	−0.42	0.07	−0.21	0.36
Personality disorder	−0.35	0.13	−0.07	0.75	−0.39	0.09	−0.34	0.14

*—insignificant after applying the Hochberg correction.

## Data Availability

Data used in this manuscript can be shared by the corresponding author upon a reasonable request.

## Abbreviations

The following abbreviations are used in this manuscript:

AMI-SF	Autobiographical Memory Inventory—Short Form
BL	Bilateral
ECT	Electroconvulsive Therapy
RAA	Retrograde Autobiographical Amnesia
RUL	Right Unilateral
TRD	Treatment-resistant Depression
